# Advanced technique for firmware security analysis through heterogeneous data fusion and knowledge mapping

**DOI:** 10.1371/journal.pone.0319660

**Published:** 2025-04-11

**Authors:** Peng Xiao, Linjiang Xie, Feilu Hang, Hanruo Li

**Affiliations:** Information Center of China Southern Power Grid Yunnan Power Grid Co., Ltd, Kunming, Yunnan, 650000, China; UNISA: Universita degli Studi di Salerno, ITALY

## Abstract

As the core component of a device, firmware’s security directly affects the stability of the entire system and the security of user data. In order to provide a more comprehensive and accurate data foundation and improve the accuracy of firmware security analysis, this article conducts research on advanced technologies for firmware security analysis through heterogeneous data fusion and knowledge mapping. Firstly, preprocess the firmware security analysis knowledge graph data using cleaning, segmentation, classification, and other processing methods. Secondly, calculate the firmware security status value under heterogeneous information based on the processed data; Again, based on the calculation results of firmware security status values, extract knowledge graph features for firmware security analysis and annotate relationship description entries; Finally, based on knowledge mapping technology, ontology integration and ontology mapping knowledge fusion were carried out to achieve more effective organization and representation of firmware security analysis knowledge, and the research on the construction technology of firmware security analysis knowledge graph was completed. The experimental results show that after applying the proposed method for firmware security analysis, the loss function index value and F1 index value are both very high, and the prediction accuracy of knowledge graph evolution is very close to 100%, with good operational effect.

## 1. Introduction

With the continuous development of the Internet, human beings have developed from a simple Web 1.0 with content acquisition and query functions to Web 2.0 that can participate in the Internet and produce content, and Web 3.0 based on knowledge interconnection. The era of the Internet of Things has made people’s lives more convenient and information acquisition faster [1]. However, the amount of firmware security related data is growing explosively. Due to the multi-source content and diverse data of the Internet, a large amount of information cannot be effectively used, which brings great challenges to security analysis [2]. The principle of knowledge organization indicates the sufficiency, orderliness and standardization of knowledge, which requires people to integrate heterogeneous and heterogeneous knowledge information on the Internet from a new perspective to meet users’ cognitive needs. With the development of technology, people’s requirements for firmware security are also increasing [3]. Firmware refers to computer programs permanently included in hardware devices, which undertake the most basic and minimum software functions in the device. Compared to other software, firmware is usually only released in binary code. Firmware manufacturers will further remove symbol information from the software during release, and perform encryption, packaging, integrity verification, and other operations for copyright and security considerations to increase the difficulty of reverse analysis [4]. The characteristics of passive code, unsigned code, and diverse instruction sets have increased the difficulty of detecting and exploiting potential attackers in firmware, which to some extent protects the security of devices [5]. However, it also leads to a relatively lax awareness of firmware security protection among manufacturers and users, which is reflected in the lack of regular or timely updates after leaks, and the lack of correctness checks for configuration items. This phenomenon also prolongs the lifecycle of security vulnerabilities contained in firmware, providing attackers with relatively sufficient time to try various attack methods. How to quickly and accurately discover security vulnerabilities from these complex data has become an important issue in the current firmware security field.

The firmware analysis method using symbol execution in reference [6], called ProXray, can learn protocol models from known firmware, apply this model to identify protocol related fields, and automatically detect functions in unknown firmware. After the training phase, ProXray can fully automate the firmware analysis process and support user queries in the form of protocol related constraints. Although ProXray can achieve a certain degree of automation, it still requires manual involvement and judgment when facing complex firmware and unknown security threats, resulting in low efficiency. In reference [7], in order to ensure the compatibility and security of smart home Internet of Things (IoT) devices, a study was conducted on the security performance of IoT device firmware. A firmware security verification mechanism was developed, which utilizes the Mask Authentication Message (MAM) protocol and a distributed file system with Inter Planetary File System (IPFS) protocol to perform secure firmware verification and updates on smart home IoT devices. Ensure decentralized communication and firmware file distribution between IoT device suppliers and IoT terminal devices, perform secure firmware updates on IoT terminal devices, and effectively safeguard the secure application of IoT firmware. However, the IPFS distributed file system requires multiple nodes worldwide to store and distribute data, which may increase the complexity and resource consumption of the system. Meanwhile, the transmission of a large amount of data in the network may also lead to network congestion issues, which cannot reflect the security level of firmware in a timely manner. Reference [8] proposes a hardware based built-in security module for the security of System on Chip (SoC), which can actively check for unsafe elements within the system, self inspect components in the SoC, and randomly conduct security tests to ensure the safe operation of firmware. However, the design of the built-in security module needs to be closely integrated with the overall architecture of the SoC and ensure seamless collaboration between the security module and other parts of the SoC. If there are flaws in the design or implementation of the security module, hackers may exploit these vulnerabilities to bypass security mechanisms and launch attacks on the SoC, seriously affecting firmware security. Although some progress has been made in the above research, in the application of firmware security analysis, due to the existence of a large amount of security information, the above methods do not consider the integration of heterogeneous and heterogeneous knowledge information on the Internet, which makes the integration of heterogeneous information still insufficient, leading to the inability to find security vulnerabilities more quickly and accurately in real applications, affecting the management effect of related enterprises.

Knowledge graph technology mainly applies mathematics, information science, and visualization techniques to divide and construct relevant knowledge content based on their relationships, and integrate them into the firmware security analysis knowledge information relationship graph [9]. For example, if you enter a keyword in your browser, it will recommend other related keywords in the search interface, and as the number of search keywords increases, it will recommend more relevant content information. As a technology that organizes large amounts of complex and unordered data in a structured manner, it provides new ideas for firmware security analysis. By constructing a knowledge graph for firmware security analysis, knowledge, concepts, entities, and their relationships related to firmware security can be represented structurally, thereby improving the efficiency and accuracy of security analysis [10]. Knowledge mapping technology can extract knowledge information from the entire Internet resources and provide users with systems and related keyword knowledge systems.

Therefore, based on knowledge mapping technology, in order to improve the effectiveness of firmware security analysis knowledge graph construction, a research on firmware security analysis knowledge graph construction technology integrating heterogeneous information is proposed. The specific technical roadmap is described as follows:

Step 1: Firmware security data often comes from different sources, with different structures and formats. Preprocessing heterogeneous information through cleaning, segmentation, classification, and other processing methods can effectively ensure the subsequent availability of its data information, thereby improving the reliability of constructing a firmware security analysis knowledge graph.

Step 2: In order to quantitatively evaluate the security status of the firmware and understand the potential security risks it may face in the current environment, the firmware security situation value is calculated to provide reliable data support for the future.

Step 3: Based on the calculation results of the firmware security situation values mentioned above, extract the knowledge graph features of firmware security analysis, and annotate the relationship description entries to gain a deeper understanding of the security status of the firmware, further improving the accuracy and efficiency of security analysis.

Step 4: In order to find security vulnerabilities quickly and accurately, in view of the wide range of data sources, the lack of deep relevance of knowledge between different data sources, and the serious duplication of knowledge, based on the above feature extraction and annotation results, considering the integration of heterogeneous and heterogeneous knowledge information on the Internet, ontology integration and ontology mapping knowledge fusion are carried out according to knowledge mapping technology to disambiguate, process, and integrate heterogeneous and diverse knowledge from different data sources, so as to achieve more effective organization and representation of firmware security analysis knowledge. Thus, the construction of firmware security analysis knowledge map integrating heterogeneous information is completed, in order to improve the management ability and work efficiency of related enterprises, and ensure firmware security.

## 2. Firmware security analysis knowledge map construction under heterogeneous information

### 2.1. Firmware safety analysis knowledge map data preprocessing

In the construction of firmware security analysis knowledge graphs, the quality and integrity of data are crucial for building efficient and accurate knowledge graphs. Therefore, in order to achieve effective construction of firmware security analysis knowledge graph, data preprocessing methods such as cleaning, segmentation, and classification are adopted to reduce invalid information, provide high-quality data for subsequent operations, and improve the efficiency of firmware security analysis knowledge graph construction. The implementation process of the preprocessing method is described as follows:

(1) Cleaning: remove invalid strings such as symbols and labels directly according to the disabled word list; For missing data such as information item blank, if it cannot be filled in effectively, remove [11]; For hypertext markup language labels, network addresses and other redundant information, it is directly removed.
(2) Word segmentation: The processing results of this stage determine the practicability and reliability of the firmware security analysis knowledge map, so the Chinese word segmentation tool of version 0.42.1 of jieba is used. Recognize the sentences scanned by prefix dictionary word map, and get the optimal state sequence through the dynamic programming calculation of Viterbi algorithm to complete word segmentation.
(3) Classification: In firmware security analysis, data often has diversity and complexity, including various types of security vulnerabilities, attack patterns, etc., and spatial information in the data is crucial for classification tasks. Convolutional neural networks can capture spatial information by performing block operations on data, which helps to identify firmware security events of different categories more quickly and accurately.

Therefore, in order to improve the quality and accuracy of subsequent knowledge graphs, a knowledge graph data classification model is established using convolutional neural networks under heterogeneous information [12,13], in order to quickly complete data classification and integration work, and improve the efficiency and accuracy of knowledge graph construction. The classification process is as follows:

① Input layer: take the segmented data results as input and input them into the input layer of the convolutional neural network;

② Convolution layer: suppose that word *τ* in sentence *m* is processed by embedded representation to obtain word vector $x_{m\tau }$, and through bidirectional recurrent neural network [14], two feature codes ${h^{\prime }}_{m\tau }$ and ${h^{\prime \prime }}_{m\tau }$ in the positive and negative directions are obtained, as shown below:


$$\{\begin{array}{l} {h^{\prime }}_{m\tau }=\vec{R_{NN}}\left(x_{m\tau }\right)\\ {h^{\prime \prime }}_{m\tau }=\overset{\leftarrow }{R_{NN}}\left(x_{m\tau }\right) \end{array}$$ (1)

③ Pooling layer: integrate attention mechanism and two-way gated recurrent neural network [15,16], build a two-level network layer, and extract features according to the weight ratio of words and sentences on semantic impact. According to the feature codes ${h^{\prime }}_{m\tau }$ and ${h^{\prime \prime }}_{m\tau }$ obtained from the convolution layer, use the tanh activation function to obtain the word vector ${x^{\prime }}_{m\tau }$ based on the attention mechanism nonlinearly, and solve its weight $M_{nb}$ through the following sigmoid activation function, which $M_{nb}$ represented as follows:


$$M_{nb}=\frac{\exp\left({x^{\prime }}_{m\tau }\right)}{\sum_{\tau }\exp\left(x^{\prime }\right)}$$ (2)

In formula (2), *x*^′^ represents any word vector integrated with attention mechanism.

Integrate heterogeneous information, generate bidirectional feature codes ${h^{\prime }}_{m\tau }$ and ${h^{\prime \prime }}_{m\tau }$ of sentence *m* according to the obtained weight $M_{nb}$ and feature codes ${h^{\prime }}_{m}$ and ${h^{\prime \prime }}_{m}$, as shown below:


$$\{\begin{array}{l} {h^{\prime }}_{m}=\sum_{m}M_{nb}{h^{\prime }}_{m\tau }\\ {h^{\prime \prime }}_{m}=\sum_{m}M_{nb}{h^{\prime \prime }}_{m\tau } \end{array}$$ (3)

④ Full connection layer: based on the bidirectional feature codes ${h^{\prime }}_{m}$ and ${h^{\prime \prime }}_{m}$ obtained above, concatenate all sentence vectors $S=\left[{h^{\prime }}_{m};{h^{\prime \prime }}_{m}\right]$;

⑤ Softmax function layer: calculate the probability distribution for each category. Assuming there are *C*categories, $Q_{i}$ represents the output vector *S* of the fully connected layer, and the input belongs to the original score of the *i*-th category. The Softmax function converts these raw scores into a probability distribution$p_{i}$, as follows:


$$p_{i}=\frac{\exp\left(Q_{i}\right)}{\sum_{i=1}^{C}\exp\left(Q_{i}\right)}$$ (4)

Finally, using the network layer, select the category with the highest probability as the classification result, and the specific output *L*is as follows:


$$L=\max\left(\sum_{i=1}^{C}p_{i}\times S+b\right)$$ (5)

In Formula (5), *b* represents the generation offset term.

Based on the above process, the data processing is completed. Next, based on the processed data, the firmware security situation value is calculated to quantitatively evaluate the security status of the firmware, in order to understand the security risks that the firmware may face in the current environment and provide reliable support for the future.

### 2.2. Firmware security situation value calculation under heterogeneous information

In the field of firmware security, the presence of heterogeneous information makes the assessment of security situations more complex. Due to numerous influencing factors, directly calculating the security situation may have a high level of complexity. Therefore, before conducting security situation calculation, it is necessary to conduct in-depth research on various influencing factors and identify the dominant factors in order to more accurately and efficiently evaluate the situational values of the dominant influencing factors of firmware and understand the security situation.

Let the sequence of each association factor set in the network node be $\left\{r_{k}\left(i\right)\right\}\left(k=0,1,\cdots ,n;i=1,2,\cdots ,l\right)$, where *n* represents the number of sub set columns and *l* represents the length of sub sequence. If the reference sequence and comparison sequence of the correlation factor set are $r_{0}$ and $r_{k}$ respectively, the difference of each factor sequence can be expressed as:


$$C_{k}=\left|r_{0}\left(i\right)-r_{k}\left(i\right)\right|$$ (6)

Then the grey correlation coefficient $R_{T}$ between $r_{0}$ and $r_{k}$ can be expressed as:


$$R_{T}=\frac{C_{\min}+C_{\max}}{C_{k}\left(i\right)+C_{\max}}$$ (7)

In Formula (7), $C_{\max}$ represents the maximum sequence difference value and $C_{\min}$ represents the minimum sequence difference value. Select the dominant influencing factors based on the set threshold $\chi_{1}$and calculate the firmware security situation value. Therefore, retain the factors where $R_{T}$ is greater than$\chi_{1}$. Then, to further ensure the reliability of the selected factors, the grey correlation entropy [17] is calculated based on the grey correlation coefficient $R_{T}$ of each factor, and its calculation formula is as follows (8):


$$Q_{k}\left(i\right)=\frac{R_{T}\left(i\right)}{\sum_{i=1}^{n}R_{T}\left(i\right)}$$ (8)

Based on the grey correlation entropy result $Q_{k}\left(i\right)$ mentioned above, further factor selection is carried out according to the threshold$\chi_{2}$, and the correlation degree sequence is obtained as $S_{k}=\left\{Q_{k}\left(i\right)\right\}$. Finally, based on the grey correlation entropy of each factor in the correlation sequence, it is converted into the security status value of the firmware. This is usually achieved by mapping gray correlation entropy to a specific numerical range (such as 0 to 1). The higher the security status value, the better the security of the firmware. The safety situation value of $S_{k}$ can be expressed as:


$$S_{k}=-\sum_{i=1}^{n}Q_{k}\left(i\right)\ln Q_{k}\left(i\right)$$ (9)

After the above calculation, the firmware security situation value under heterogeneous information is obtained, and the firmware security analysis knowledge map features are extracted according to the value.

### 2.3. Feature Extraction of Firmware Safety Analysis Knowledge Map

**Fig 1:**
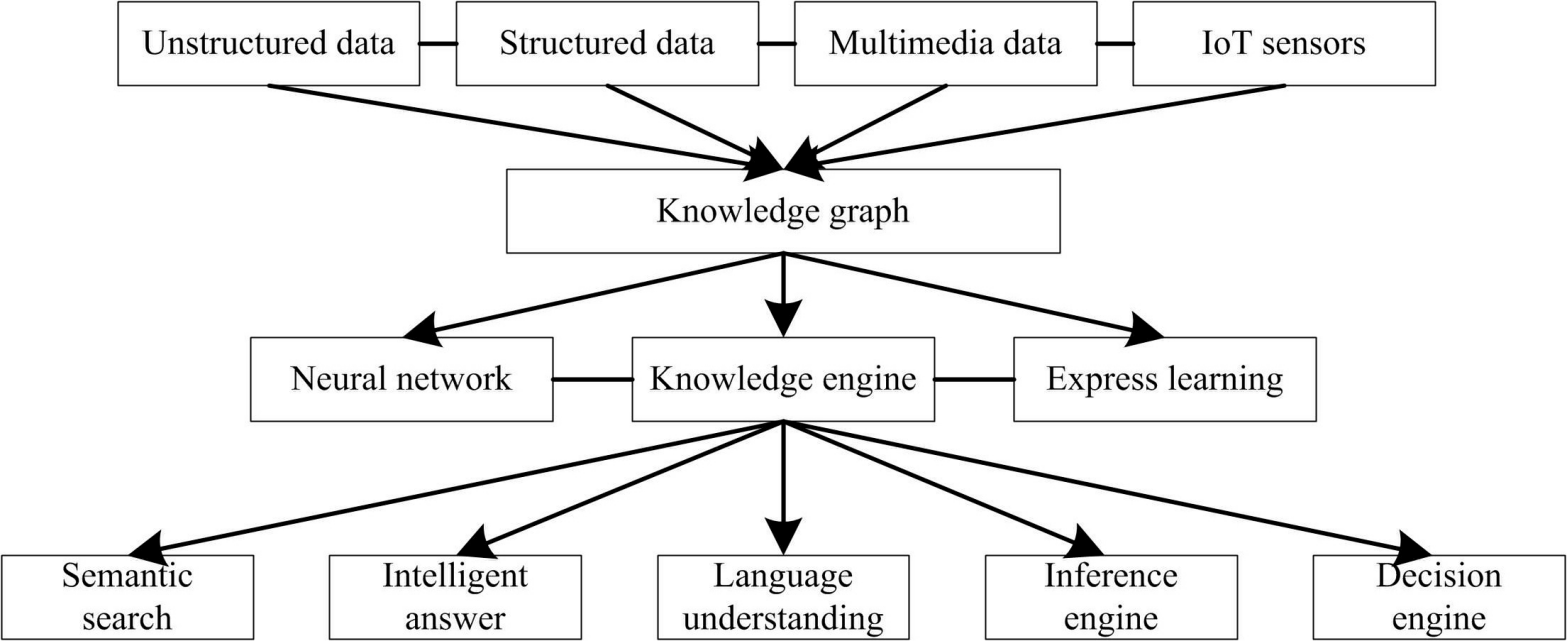
Semantic Network Diagram of Firmware Security Analysis Knowledge Map.

The concept of firmware security analysis knowledge map is a typical multilateral relationship diagram, which is composed of nodes (entities) and edges (relationships between entities). Firmware security analysis knowledge map is essentially a semantic network, which is used to reveal the relationship between everything, as shown in Fig 1.

According to Fig 1, the firmware security analysis knowledge graph is a computable model aimed at extracting concepts, entities, and their interrelationships from various complex data types. This knowledge graph can be divided into two categories based on its coverage of knowledge and application fields: general firmware security analysis knowledge graph and domain specific firmware security analysis knowledge graph. The general knowledge graph covers a wide range and is applicable to multiple domains, while the domain specific knowledge graph focuses on the firmware security analysis requirements of a specific domain. Through this approach, knowledge graphs can effectively organize and represent firmware security related information, providing support for analysis and decision-making. With the continuous development of science and technology, the firmware security analysis knowledge map has been widely used in the NLP field [18], such as semantic search, intelligent question answering, decision-making assistance, etc., which has become an important driving force for the development of artificial intelligence.

A common representation of the firmware security analysis knowledge map is the triple form, namely:


$$G=\left(g_{1},g_{2},g_{3}\right)$$ (10)

In Formula (10), $g_{1}$ represents the head entity in the triple *G*, $g_{3}$ represents the tail entity in the triple *G*, and $g_{2}$ represents the relationship between the two entities.

**Fig 2:**
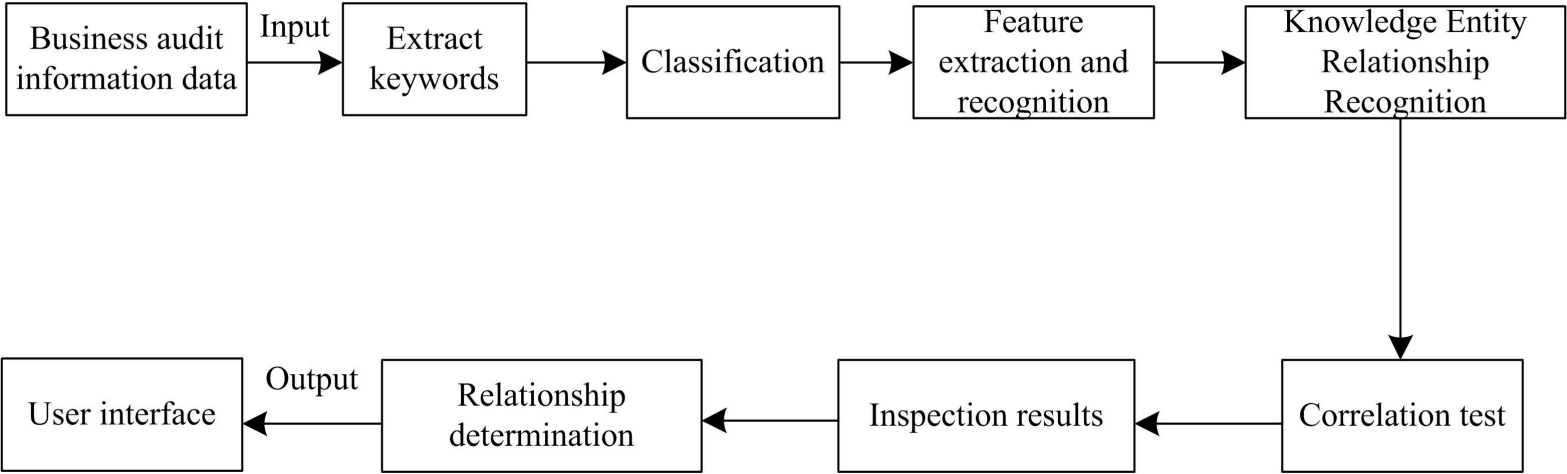
Firmware security analysis knowledge map processing system flow.

At the same time, the firmware security analysis knowledge map also has the inspection function, which can inspect and analyze the business content according to its own retrieval rules, automatically point out errors, modify and correct the content with problems, intelligently analyze the original information of the audit work order and the verified reason description, propose feedback information such as audit verification steps and rectification measures guidance, and determine the type of causes of the exception, It also marks the cause tag of the problem, assists the business personnel in their work, improves the efficiency of the creation, maintenance and management of audit rules, and thus realizes the intellectualization of business control support. Conduct module operation training on the knowledge map. Fig 2 shows the operation process of the firmware security analysis knowledge map processing system:

Transfer information resources such as business work order content to the knowledge map processing system in Fig 2, carry out vocabulary extraction, classification and feature extraction, and obtain the description of the relationship between vocabulary and language according to its features and related descriptions, and mark them; Then, the information dataset is checked to check the correctness of the business rule information content and determine whether there is a problem with the work order information. The information content is determined by the degree of association between different documents or words according to the keywords and relationship description of the knowledge map system. If there is a problem, the reason for the problem is marked; Finally, the inspection results are output to the user display interface.

**Fig 3:**
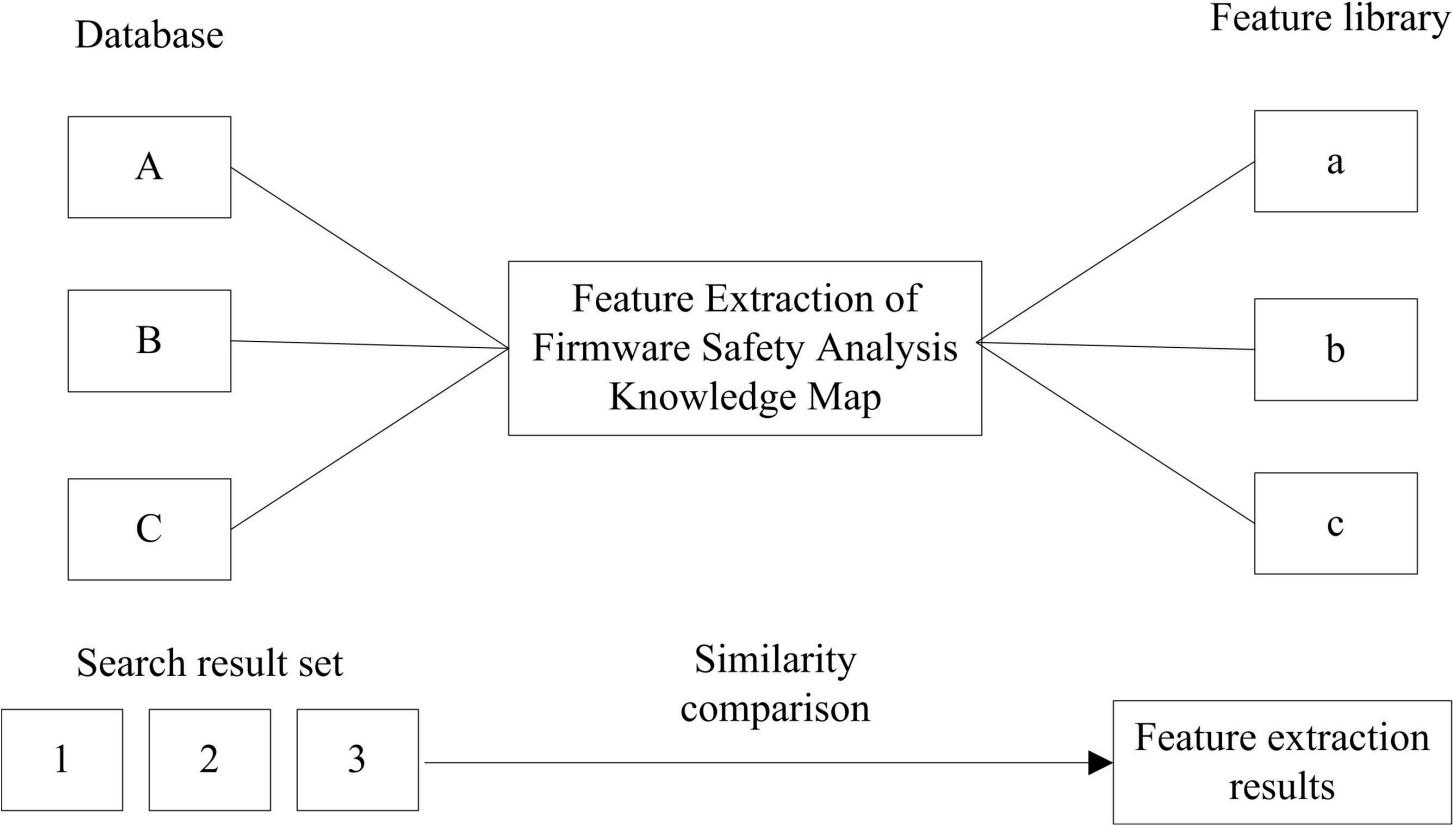
Feature Extraction Results.

Heterogeneous information is fused to identify and judge the relationship of key words, and the feature extraction results are shown in Fig 3:

According to Fig 3, the acquired knowledge entity vocabulary and its associated vocabulary and documents are used for language processing and relationship recognition. As the analysis object, knowledge entity language selects different recognition methods according to the description features, such as character features, part of speech features, meaning content, etc. More specific identification can be carried out according to the classification of relevant words, such as person name, place name, organization name, professional working vocabulary, etc. In the knowledge map of firmware security analysis, key words or languages mainly include problem objectives, problem descriptions, fast output topics matching objectives, business control rules, etc.

According to the description features, we can identify and analyze the words and sentences that have an association relationship, infer the nature of the relationship between them, define and describe the relationship, and add the interpretation of the association terms. At the same time, the documents of both parties also participate in the construction of the relationship map of these two words or languages; For the relationship identification of document content, start with the correlation degree of keywords of both parties, combine the identification results and relationship determination results of other languages to describe the relationship to construct the association relationship, and annotate the relationship description entries to complete the feature extraction of the firmware security analysis knowledge map.

### 2.4. Firmware security analysis knowledge map construction technology

The construction of firmware security analysis knowledge map needs to be applied to various information processing technologies. Knowledge extraction extracts knowledge from a variety of data sources and stores it in the firmware security analysis knowledge map, which is the basis for building a large-scale firmware security analysis knowledge map. Knowledge fusion can solve the heterogeneous problem of different firmware security analysis knowledge maps. Through knowledge fusion, heterogeneous knowledge maps of different data sources can be interconnected and interoperated, thus improving the quality of firmware security analysis knowledge maps. Knowledge computing is the main output capability of knowledge map, among which knowledge reasoning is one of the most important capabilities, which is the realization way of knowledge refinement and auxiliary decision-making.

The data sources are very extensive, the knowledge between different data sources lacks in-depth correlation, and the problem of knowledge duplication is very serious. Knowledge fusion will disambiguate, process and integrate heterogeneous and diversified knowledge from different data sources under the same framework to achieve the fusion of data, information and other aspects. The core of knowledge fusion is the generation of mapping.

**Fig 4:**
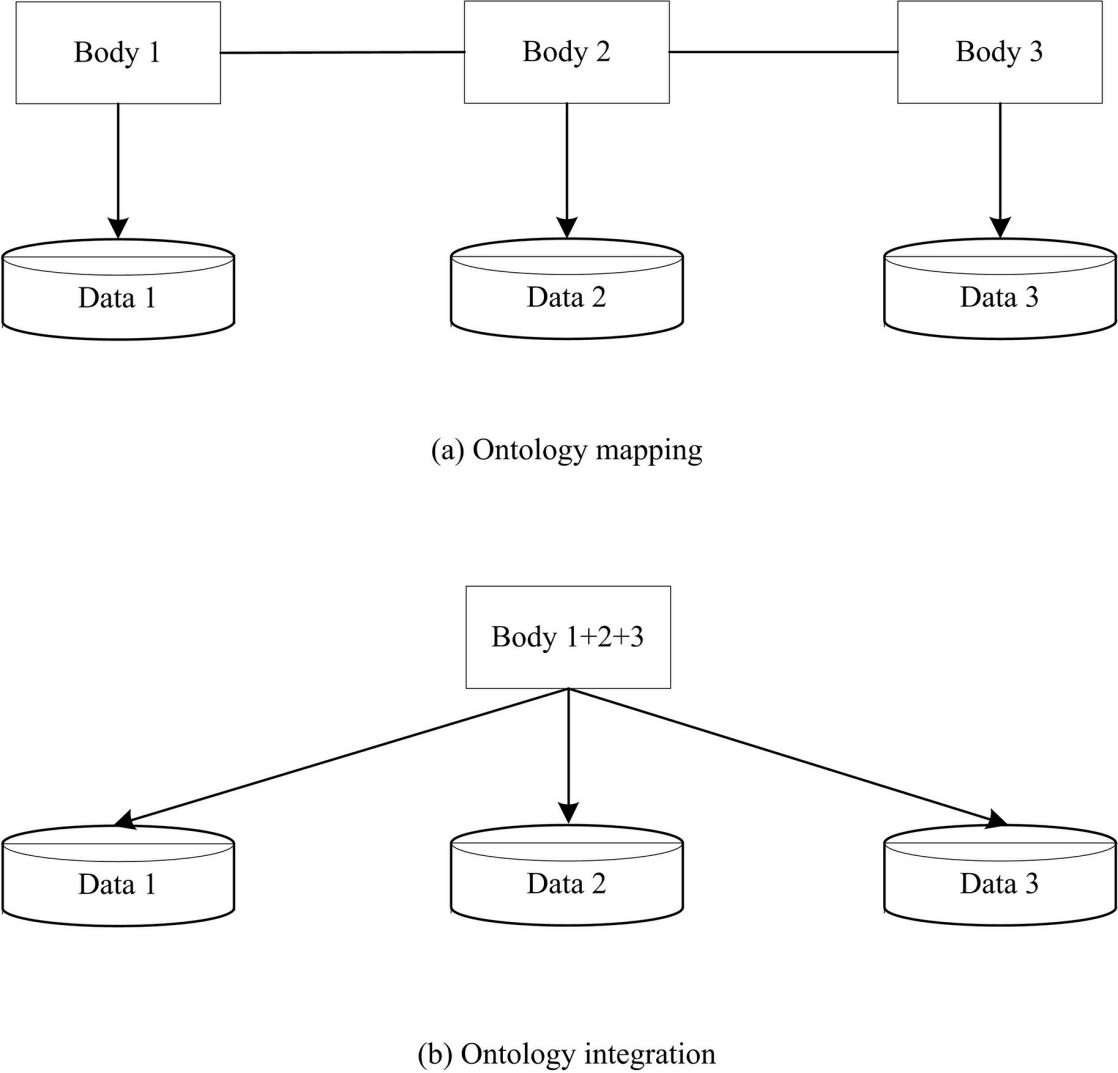
Structure of ontology mapping and integration.

In knowledge fusion technology, ontology layer occupies an important part. It is mainly divided into ontology integration and ontology mapping, as shown in Fig 4.

According to Fig 4, ontology integration is to integrate heterogeneous ontologies from multiple different data sources into a unified ontology, and ontology mapping is to establish mapping rules among multiple ontologies to transfer information between different ontologies.

**Fig 5:**
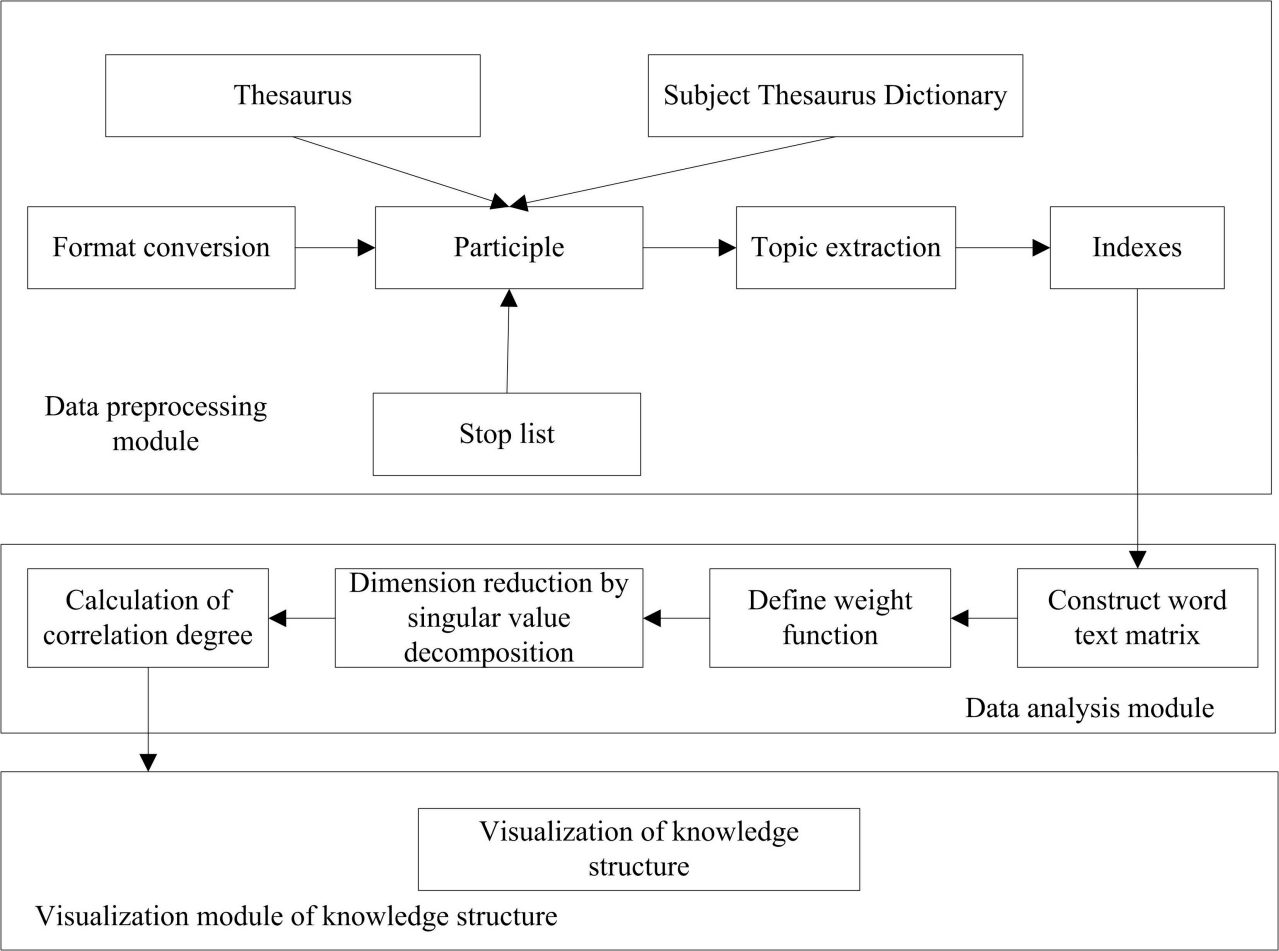
Structure of Firmware Security Analysis Knowledge Map.

Use the above structure to select appropriate data objects, and use them as the firmware security analysis knowledge map to build a text data source, and standardize the data source. Based on the processed data, the latent semantic analysis is carried out, and the XTM visualization map is drawn according to the results, so as to complete the construction of the firmware security analysis knowledge map. Fig 5 is the structure diagram of firmware security analysis knowledge map.

**Fig 6:**
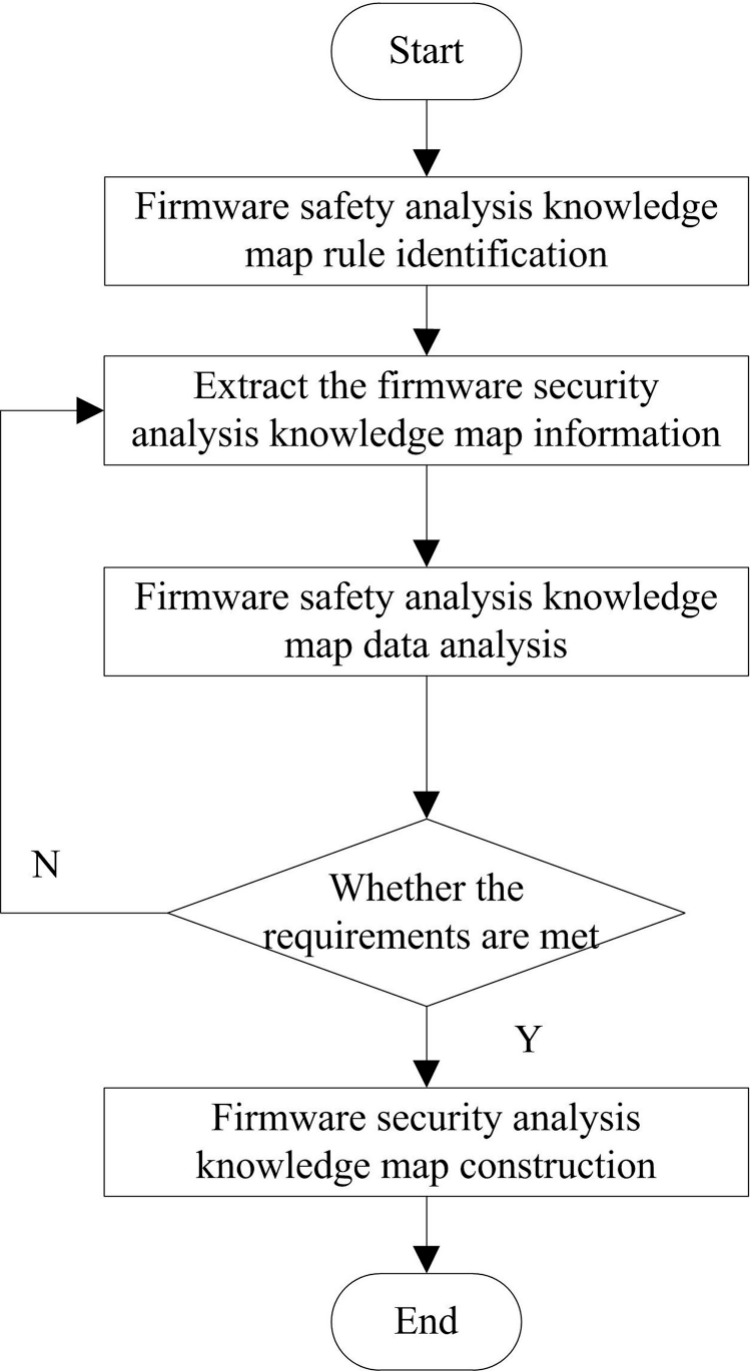
Firmware security analysis knowledge map construction process.

The process of building a firmware security analysis knowledge map includes two main steps: knowledge element identification and relationship analysis. The workflow of building a firmware security analysis knowledge map that integrates heterogeneous information is shown in Fig 6:

The detailed process of building the firmware security analysis knowledge map is as follows:

Step 1: For the preprocessed text, generate the corresponding word text matrix. Based on the relationship table in the firmware security analysis knowledge map database, realize the dimension merging of the same word, and reduce the dimension of the matrix to obtain the word text matrix.

Step 2: Singular value decomposition under latent semantic analysis is used to reduce the matrix dimension twice, and high-frequency words are extracted as candidate feature items.

Step 3: Filter the candidate feature items by using the subject term in the database. If the term is included, the candidate tag will be retained and discarded instead.

After completing the construction of ontology mapping, ontology integration structure and firmware security analysis knowledge map structure, integrate its relationship processing data resources and build the firmware security analysis knowledge map process. Divide the analyzed and processed knowledge entity information and related data according to a certain relationship, import them into the database of the model building system in batches, and the system uses Cypher language to program the framework of the vocabulary and document relationship model. Cypher can query the associated nodes and key descriptions of all relationship features of vocabulary or documents in the system resource database and Internet platform, further improve the relationship network and associated description between vocabulary based on the retrieved relationship results, push forward layer by layer, and build an interrelated relationship network. At the same time, Cypher can also make personalized judgments on its relevance according to the queried relationship information, and build a relationship network with different tightness according to the degree of relevance. Therefore, when users use this knowledge map, the system will recommend information content with high relevance according to the relevance of search keywords, and the number of other recommended content will decrease according to the relevance, so that users can enjoy more personalized and intelligent retrieval services. In addition, the atlas is in synchronous contact with the system database, which will make relevant records of the retrieval content, update and enter new information resources at any time, maintain the real-time nature of the knowledge atlas, and ensure the authenticity and integrity of the relevant work content records, thus completing the research on the construction technology of the firmware security analysis knowledge atlas integrating heterogeneous information.

## 3. Experimental analysis

In order to verify the effect of the firmware security analysis knowledge map construction technology that integrates heterogeneous information, an experiment is designed. The experimental environment is as follows: the development platform is Eclipse, the ontology development platform is Protege4.3.0, the programming language is Jave, OWL, JavaScript, the database is Mysql, the word segmentation tool is NLPIR system, and the server is Apache Tomcat tool. The experimental dataset is defined as Common Crawl unstructured data A, JSONPlaceholder semi-structured data B, and UCI Machine Learning repository structured data C. Among them, dataset A contains all formats of office documents, text, images, HTML, various reports, images, and audio/video information, etc. The data structure is irregular and there is no predefined data model. Dataset B is JSON format data with standard format definitions, consisting of objects and arrays, and supports Schema Evolution, which means that the data structure can change with business requirements without modifying the underlying database table structure. The data in dataset C is stored in a two-dimensional table consisting of rows and columns, with a strict data structure that follows a predefined data model. Other experimental parameters are shown in Table 1.

**Table 1:** Statistics of specific hardware and software environment parameters of the experiment.

Experimental environment	Parameter name	Parameter content
Hardware environment	RAM	8GB
	Processor	Intel Core i3
	ROM	1TB
Software environment	Vector Conversion Tool	Word2Vec
	Operating system	Windows7
	Visualizer	Echatrs
	Compiler	Gcc4.3.0
	Integrated development environment	My Eclipse
	Database	MySQL5.7
	Development kit	Jena、Scraoy1.0

In order to improve the experimental reliability of firmware security analysis knowledge map construction technology integrating heterogeneous information, it is assumed that the real item and identification item are *λ* and *λ*^′^ respectively, and the norm form of the two contrast items is ${\left\|\lambda |\lambda^{\prime }\right\|}^{2}$ The greater the $J\left(\lambda ,\lambda^{\prime }\right)$ value of the number loss function, the more accurate the knowledge map of the model construction; $J\left(\lambda ,\lambda^{\prime }\right)$ is shown in Formula (11).


$$J\left(\lambda ,\lambda^{\prime }\right)=-\frac{1}{m}\sum_{Z=1}^{m}Z_{Z}\log\left(J_{Z}\right)+{\left\|\lambda |\lambda^{\prime }\right\|}^{2}$$ (11)

In Formula (11), *m* represents the number of samples, $Z_{Z}$ represents *Z* samples that the model predicts as positive cases and are actually positive cases, and $J_{Z}$ represents *Z* samples where the model predicts a positive case but actually a negative case.

The accuracy of technology is negatively correlated with the logarithmic loss function index, and the effectiveness of technology is positively correlated with the F1 index. The higher the F1 value, the better the model performance. The solution formula of FF index is shown in Formula (12).


$$F1=\frac{2\times Z_{pre}\times Z_{rec}}{Z_{pre}+Z_{rec}}$$ (12)

**Fig 7:**
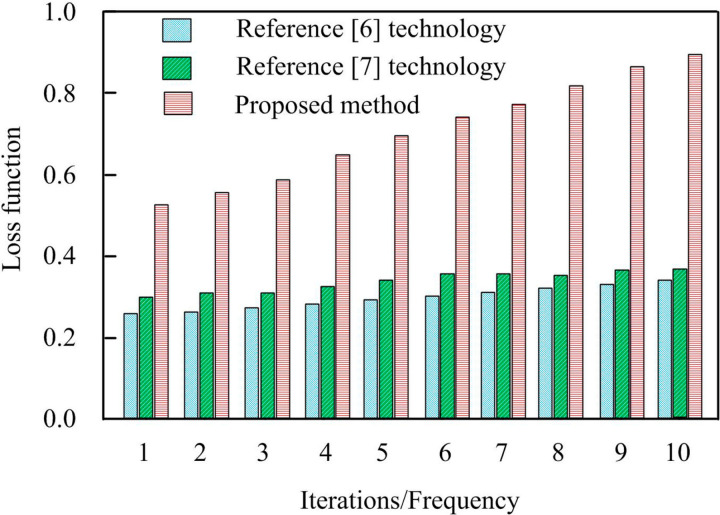
Trend Results of Loss Function Curve.

**Fig 8:**
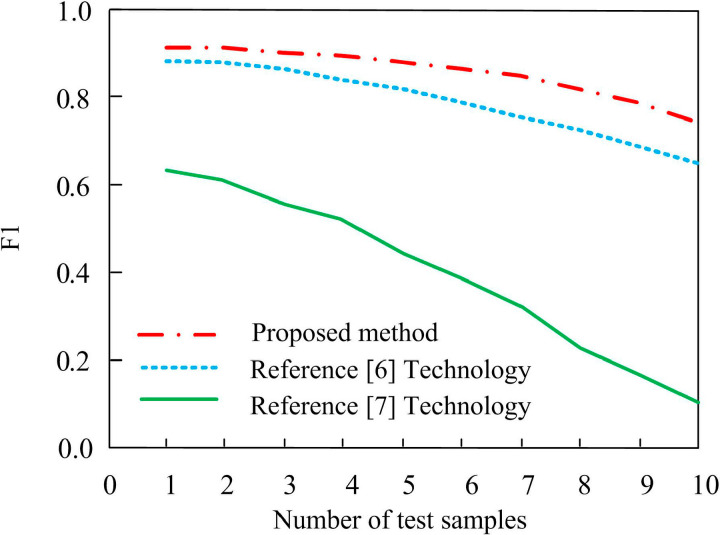
Trend Results of F1 Index Value Curve.

In Formula (12), $Z_{pre}$ represents the accuracy rate and $Z_{rec}$ represents the recall rate. According to the above formula, the evaluation diagram of the knowledge entity recognition effect of different methods is shown below. The trend of the loss function under different methods varies with the number of iterations, as shown in Fig 7. The curve trend of F1 index value with the number of iterations is shown in Fig 8.

According to Fig 7, there is a certain linear relationship between the index value of the loss function of this technology and the number of iterations. The rise and fall range is large and the speed is fast; While the reference [6] technology and reference [7] technology not only have the index values always been far lower than the technology in this paper, but also have a small rise and fall range. This indicates that after a certain number of iterations, the value of the loss function of this technique is larger, indicating that the firmware security analysis knowledge graph constructed by it is more accurate. This is because the technology in this article processes the data before constructing the knowledge graph, providing higher quality data for the construction of the knowledge graph, thereby better learning key information and patterns related to firmware security, and more effectively capturing complex relationships in the data during the iteration process, thereby achieving higher accuracy in firmware security analysis tasks.

From Fig 8, it can be seen that the F1 index value of this technology is consistently higher than that of the reference [6] technology and the reference [7] technology. When the number of test samples reaches 10, the F1 index value of the technology in this article can still be maintained above 0.8. The F1 index values of reference [6] technology and reference [7] technology are below 0.7 and 0.6, respectively. Therefore, comparing the results obtained from the above three technologies, it can be seen that the knowledge graph constructed by this technology is more reliable and can effectively ensure the accuracy of firmware security analysis. The reason is that this technology uses ontology mapping and ontology integration structure to select appropriate data objects, which are used as firmware security analysis knowledge maps to build text data sources, and to standardize the processing of data sources. Based on the processed data, potential semantic analysis is carried out, XTM visualization map is drawn according to the results, and firmware security analysis knowledge map is constructed, which is beneficial to the trend of F1 index value curve to some extent.

**Fig 9:**
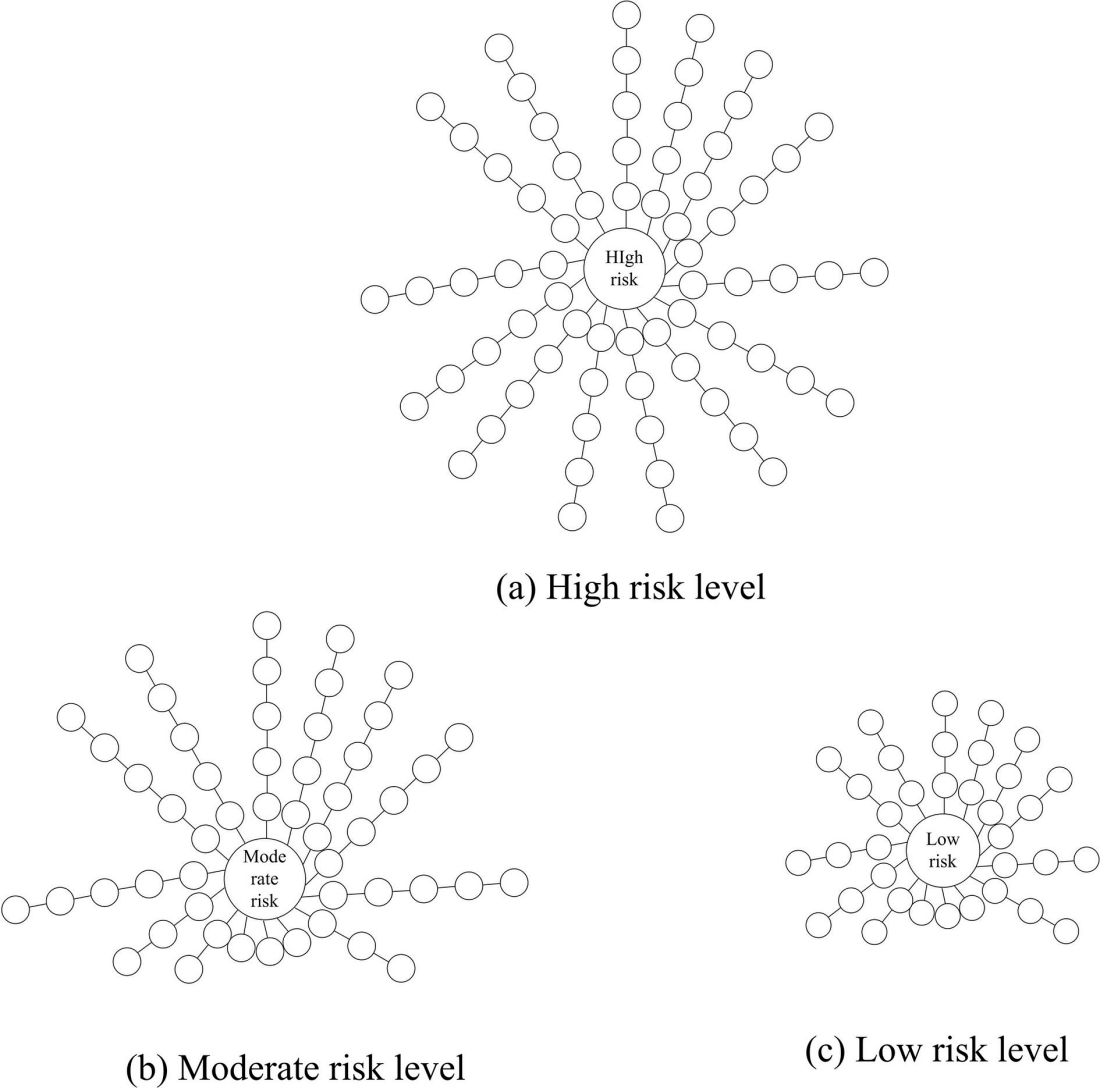
Firmware Security Analysis Knowledge Map Vulnerability Hazard Level.

Integrate heterogeneous information, and use the firmware security analysis knowledge map to conduct multi-dimensional analysis of vulnerability types. The firmware security analysis knowledge map has three types of vulnerability hazard levels, high, medium and low, as shown in Fig 9.

**Fig 10:**
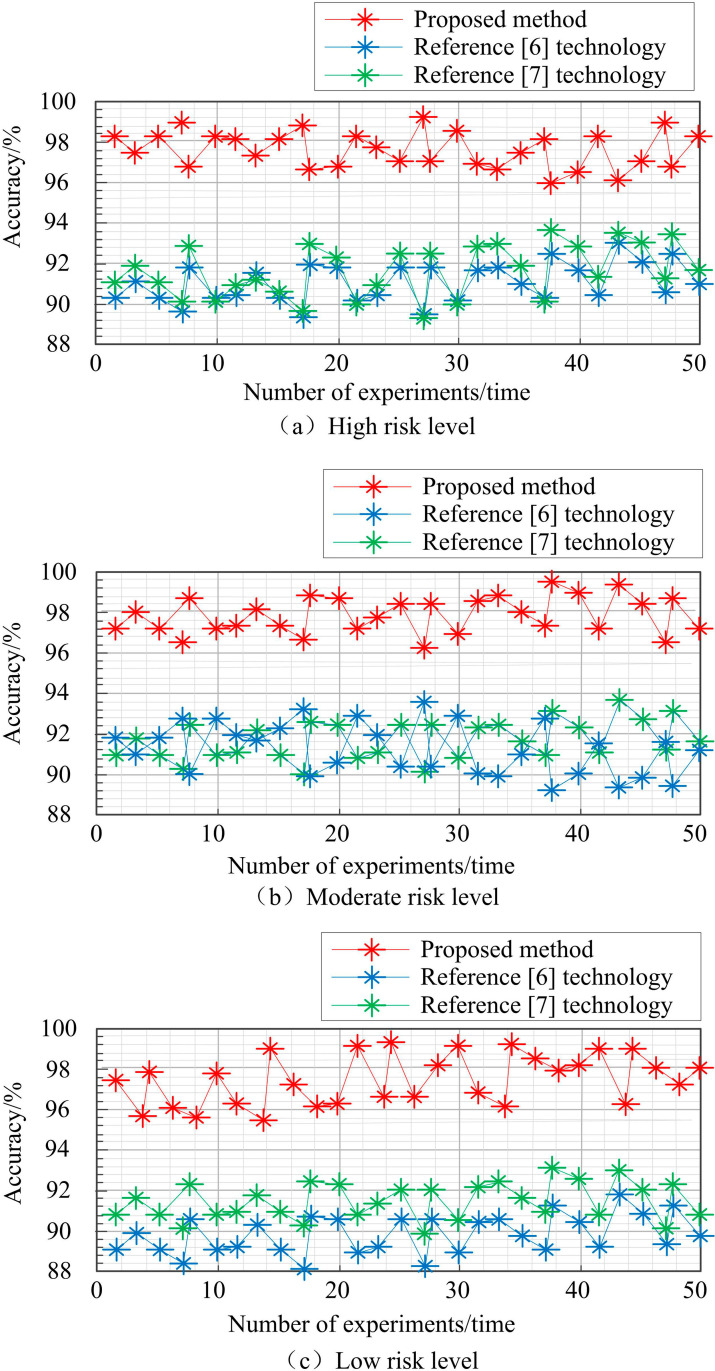
Prediction evolution accuracy of knowledge map.

It can be seen from Fig 9 that, through the firmware security analysis knowledge map, the vulnerability hazard level is integrated with heterogeneous information, and the security event nodes are traversed. When the field values exist at the same time, the values are mapped to the event ID, and the vulnerability levels in all three cases are spread around. Keep the above experimental environment unchanged, and verify the prediction evolution accuracy of firmware security analysis knowledge map under this technology. The results are shown in Fig 10.

The analysis in Fig 10 shows that under different vulnerability hazard levels, the proposed method’s knowledge graph has the highest predicted evolution accuracy, very close to 100%, demonstrating good operational performance. Among them, under high-risk vulnerabilities, the predicted evolution accuracy of the method in reference [6] is 89.2-93.2%, the predicted evolution accuracy of the method in reference [7] is 88.5-93.5%, and the predicted evolution accuracy of the proposed method is 96.1-99.5%; Under the vulnerability of medium hazard level, the predicted evolution accuracy of the method in reference [6] is 88.8-93.5%, the predicted evolution accuracy of the method in reference [7] is 90.1-93.7%, and the predicted evolution accuracy of the proposed method is 96.2-99.8%; Under high-risk vulnerabilities, the predicted evolution accuracy of the method in reference [6] is 88.2-91.8%, the predicted evolution accuracy of the method in reference [7] is 89.8-93.1%, and the predicted evolution accuracy of the proposed method is 95.5-99.2%. The predicted evolution accuracy of the proposed method is consistently higher than the other two methods, and higher than 95%. This is because in the process of constructing a knowledge graph, this technology standardizes the collected data sources through text format conversion, word segmentation, feature extraction, etc., effectively improving the quality of the data and providing reliable support for graph construction. In addition, considering the integration of heterogeneous and heterogeneous knowledge information on the Internet, the knowledge fusion of ontology integration and ontology mapping is carried out according to the knowledge mapping technology, disambiguation, processing and integration of heterogeneous and diverse knowledge from different data sources are carried out, realizing more effective organization and representation of firmware security analysis knowledge, thus further improving the accuracy of knowledge map prediction evolution.

## 4. Conclusion and prospect

### 4.1. Conclusion

(1) This study proposes an advanced technology approach that combines heterogeneous data fusion and knowledge mapping in the field of firmware security analysis. Preprocess knowledge graph data related to firmware security to improve data quality; Based on preprocessed data, calculate the security status value of firmware in heterogeneous information environments; Extract key knowledge graph features based on safety status values and annotate relationship descriptions in detail to enrich and clarify knowledge expression; By utilizing knowledge mapping technology to achieve ontology integration and knowledge fusion, the organizational structure and representation of firmware security analysis knowledge were optimized, and a knowledge graph for firmware security analysis was successfully constructed. The loss function index value and F1 index value of this technology are linearly related to the number of iterations, with large increases and decreases and fast speed. Through firmware security analysis knowledge graph, integrate vulnerability risk levels with heterogeneous information, and traverse security event nodes. The degree of vulnerability in all three scenarios spreads to all parties involved. The predicted evolution accuracy of the proposed technical knowledge graph is very close to 100%, demonstrating good operational performance. This study not only improves the efficiency and accuracy of firmware security analysis, but also provides new ideas and methods for knowledge management and application in related fields.
(2) Traditional firmware security analysis methods often rely on a single data source or specific types of data, making it difficult to fully reflect the firmware security status. And in terms of knowledge representation, it may be relatively scattered, lacking a unified knowledge modeling and representation framework. This article proposes a new firmware security analysis framework through heterogeneous data fusion and knowledge mapping techniques, which can more effectively process and integrate data from different sources, thereby improving the efficiency and accuracy of firmware security analysis. This is crucial for timely detection and repair of security vulnerabilities in firmware, helping to reduce system risks and losses caused by firmware security issues. With the rapid development of technologies such as the Internet of Things and embedded systems, firmware security issues are becoming increasingly prominent. The research results of this article will help promote the development of firmware security analysis related industries and provide technical support and services for related enterprises.

### 4.2. Prospect

This paper has made some achievements in applying heterogeneous information to the construction of firmware security analysis knowledge map. However, based on the summary and analysis of the research results of this paper, the current research on knowledge mapping technology and the research on the development characteristics of the firmware security analysis field, the research on the construction technology of firmware security analysis knowledge mapping mainly needs to be further developed and improved in the following aspects:

(1) In the next step, we can design a method that combines traditional machine learning methods with deep learning methods in the concept extraction phase, automatically compare the extraction effect according to the corpus size, select an appropriate training model for concept extraction, and further improve the extraction results.
(2) In the next step, we can consider the extraction algorithm of more types of relationships in the relationship extraction stage, expand the scale of recognizable relationships, try to extract relationships based on deep learning, try to integrate the extraction methods of multiple relationship types into a common extraction model, and further improve the accuracy and recall of relationship extraction.
(3) The traditional knowledge extraction task uses the flow pipeline mode. The task is divided into two modules named entity recognition and relationship extraction. The relationship extraction is completed on the results of entity recognition. This method may lead to error propagation and amplification. Therefore, how to make full use of the internal relationship between entities and relationships to achieve joint extraction of firmware security entity relationships needs further research.
(4) Due to the extraordinary speed of data change in the firmware security field, the knowledge update iteration speed is very fast, but limited by the scope and frequency of firmware security data collection, the capability of knowledge mapping is not maximized. Therefore, how to use knowledge reasoning technology to improve the reasoning ability of knowledge map itself and realize the intelligent evolution of firmware security knowledge will be the next research focus.
